# Unravelling superstructure and electronic ordering in LiNiO_2_ bulk single crystals grown by optical floating zone technique

**DOI:** 10.1107/S2052520626001009

**Published:** 2026-03-05

**Authors:** Uthayakumar Sivaperumal, Daniel G. Porter, David J. Voneshen, Jon P. Goff

**Affiliations:** a Department of Physics Royal Holloway, University of London Egham TW20 0EX UK; b ISIS Pulsed Neutron and Muon Source STFC Rutherford Appleton Laboratory Didcot OX11 0QX UK; c Diamond Light Source Harwell Science and Innovation Campus DidcotOX11 0DE UK; University of Warwick, UK

**Keywords:** single-crystal growth, floating-zone technique, superstructure, single-crystal XRD, REXS

## Abstract

Although there have been several studies on the powder form, there has yet to be a report on the bulk crystal growth and its property studies of LiNiO_2_. In the present study, we report the first successful growth of a LiNiO_2_ single crystal by employing the optical floating-zone technique. Structural properties have been studied using single-crystal X-ray diffraction (XRD). The structural refinement of the single-crystal XRD data, along with the Laue diffraction patterns, confirms that this system crystallizes in a rhombohedral unit cell in space group *R*3*m* and the presence of a single grain along the length of the grown crystal. Furthermore, for the first time, we have observed and determined their superstructures as a function of temperature using single-crystal XRD. We have also conducted a study using the high flux of synchrotron X-rays to demonstrate the mechanism which drives the superstructure witnessed by the single-crystal XRD. Resonant elastic X-ray scattering was used to confirm the superstructure and the mixed valence state of different Ni sites. No additional ordering phenomena were observed, including magnetic or electronic ordering. Our study demonstrates the optimization of LiNiO_2_ growth parameters and provides information about atomic and electronic ordering in the system, including the onset of a superstructure phase. This will provide a basis for further work in developing improved cathode materials and understanding quantum spin liquids.

## Abstract 2

A breakthrough in the first successful single-crystal growth of LiNiO_2_ using the floating zone technique is reported. The as-grown single-crystal quality has been thoroughly analysed using laboratory and high-energy X-rays. Superlattice reflections are found, clearly seen using the laboratory diffractometer and resonant elastic X-ray scattering.

## 1. Introduction

Ni-rich layered oxides are promising cathode materials for Li-ion batteries since they fulfil the energy density requirements of the automobile industry (Andre *et al.*, 2015 ▸). They offer capacities higher than 180 mA h g^−1^ (commercial Li-ion batteries have capacities of 140 mA h g^−1^) at relatively high mean voltages of 3.6–4.0 V with outstanding efficiencies and sufficient power densities, but they tend to degrade relatively fast. In these rhombohedral structures, transition metals and Li ions occupy edge-sharing oxygen octahedra in alternating layers. However, the magnetic and electronic properties of the materials have been under discussion since their discovery, though this knowledge is essential in understanding their functionality and the reasons for failure upon long-term operation (Kleiner & Ehrenberg, 2017 ▸).

Immense research has been made not only based on the exceptional electrochemical performance of LiNiO_2_, but also this system is thought to be a potential candidate for its quantum spin liquid behaviour. Initially, LiNiO_2_ was proposed as a model material for an *S* = ½ triangular antiferromagnet with Ising-like interactions. The ground state was theorized to be a quantum spin liquid predicted by Fazekas & Anderson (1974 ▸). A neutron experiment conducted at the ISIS spallation source indicated a broad range of magnetic excitations, suggesting a complex behaviour (Hirakawa *et al.*, 1990 ▸). However, several previous powder neutron measurements have not detected any long-range order in LiNiO_2_ (Hirakawa *et al.*, 1985 ▸; Yoshizawa *et al.*, 1990 ▸), and a semi-disordered cluster model has been proposed based on high-field magnetization measurements (Chappel *et al.*, 2002 ▸). Although there have been several studies on the powder form, there has yet to be a report on the bulk crystal growth and characterization of LiNiO_2_.

Until now, the solid-state reaction method has been the most widely used way to synthesize LiNiO_2_. The high activation energy of this technique made it difficult to maintain stoichiometry during the calcination and sintering process. This resulted in particle coarsening and generated stress and strain, and eventually led to crack formation during the electrochemical cycling process. As a consequence, it significantly impaired the electrochemical properties, and it was outperformed by single crystals (Choi *et al.*, 2025 ▸). In our earlier studies, we demonstrated the efficacy of a floating zone technique for the single-crystal growth of battery materials (Uthayakumar *et al.*, 2014 ▸; Voneshen *et al.*, 2013 ▸; Willis *et al.*, 2018 ▸; Porter *et al.*, 2014 ▸), and we strongly believe our experience in overcoming the high volatility of Li in the growth of single crystal LiCoO_2_ can help to optimize the growth conditions of LiNiO_2_. Hence, we employed the four-lamp floating zone technique for the single-crystal growth of LiNiO_2_.

## 2. Crystal growth

**Figure 1:**
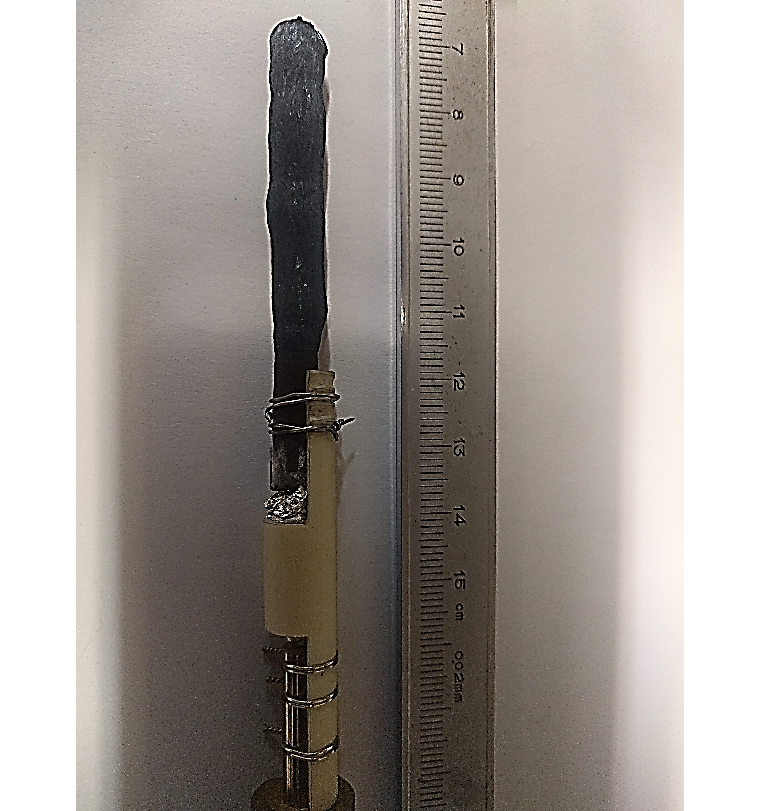
As-grown single crystal of LiNiO_2_.

A single crystal of LiNiO_2_ was grown using the four-lamp optical floating zone technique. To begin with, single-phase polycrystalline LiNiO_2_ was prepared from high-purity Li_2_CO_3_ and NiO starting materials, mixed in a ratio of 1.2:2. During the growth of LiNiO_2_ single crystals, volatilization of lithium caused considerable challenges. An excess of 20% Li_2_CO_3_ was used to counteract the volatility during the crystal growth process. Before weighing, the precursors were preheated at 823 K for 15 h to remove the moisture. After grinding the stoichiometric powders in an agate mortar for an hour, the mixture was calcined in air at 1003 K–1023 K for 24 h with intermittant grinding. Finally, the powders were compressed in the form of cylindrical rods (8 mm diameter and 12 cm length) and sintered at 1053 K for 15 h under an atmosphere of flowing oxygen. The crystal growth was performed in a four-lamp optical furnace containing a high-pressure (8.5 atm) oxygen-rich atmosphere with a translational speed of 2–5 mm h^−1^, and both feed and seed rods were counter-rotated at 10–15 rpm. The as-grown single crystal is shown in Fig. 1 ▸. This single crystal was subjected to various characterizations.

A Rigaku Synergy diffractometer with a molybdenum source at STFC Materials Characterization Laboratory was used to study the crystal structure of the sample, together with a Cryojet system for temperature control. The reciprocal space surveys allowed us to determine the propagation vector and symmetry of the superlattice reflections. At each temperature, an extensive coverage of reciprocal space was obtained to a resolution of 0.4 Å. Reflections were indexed and integrated using the standard *CrysAlisPro* (Rigaku Oxford Diffraction, 2018 ▸) software, and refinements were performed using *Jana2006* (Petříček *et al.*, 2014 ▸).

Although neutron powder diffraction is the most common approach for magnetic structure studies, it needs additional complementary tools to resolve a magnetic structure completely (Uthayakumar *et al.*, 2025 ▸). As a promising alternative, resonant elastic X-ray scattering (REXS) is a versatile synchrotron X-ray technique that offers the unique advantage of being element and shell-specific. This feature facilitates positional and occupational information about the atoms present in a crystal (Bereciartua *et al.*, 2025 ▸). In the present study, REXS measurements were conducted at the Materials and Magnetism Beamline I16 at Diamond Light Source (Collins *et al.*, 2010 ▸). The I16 six-circle kappa-geometry diffractometer provides control of the scattering plane in reciprocal space relative to the polarization axis, allowing azimuthal scans and grazing-incidence diffraction. From the as-grown crystalline boule of LiNiO_2_, individual crystals were cleaved and screened using X-ray diffraction to find a crystallite with a large, shiny surface with strong reflections along the [00*l*] direction. Such a crystal with high reflectivity was selected and mounted inside the I16 cryostat under vacuum to minimize oxidation. Reciprocal space maps were performed with the Pilatus3 100 K area detector at room temperature and the cryostat base temperature (6.5 K) to look for superlattice reflections and any evidence of magnetic order. Once the propagation vector was known, several reflections were measured to generate a complete reflection list. The incident energy was tuned to the Ni *K* edge and energy scans were performed on a number of reflections to look for any evidence of resonant scattering. A reciprocal space map was made at multiple energies round the Ni *K* edge to look for any evidence of long-range electronic ordering effects.

## 3. Results and discussion

**Figure 2:**
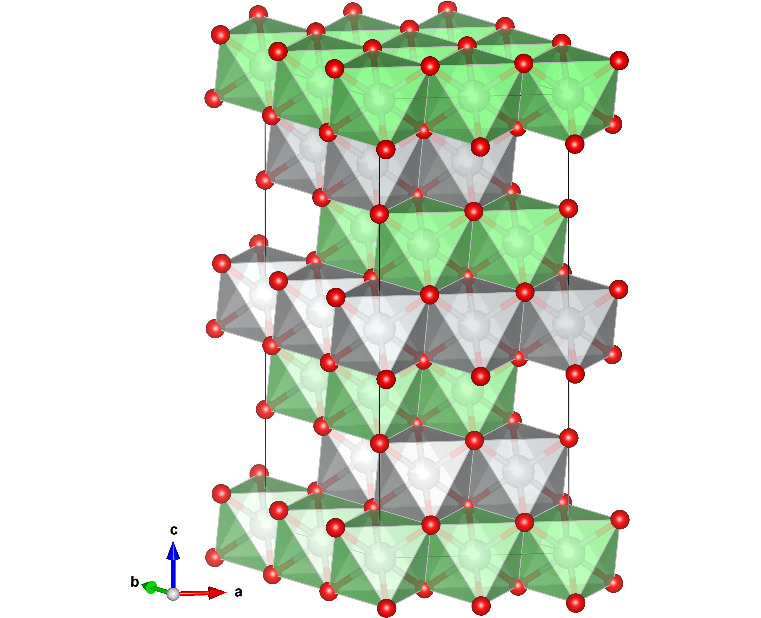
2 × 2 superstructure of LiNiO_2_.

The as-grown 8 cm crystalline boule (Fig. 1 ▸) was cleaved to find crystallites with strong reflection intensities and no other crystalline components. These were then measured with longer exposures and greater coverage at several temperatures. Single-crystal XRD confirms LiNiO_2_ crystallizes in space group *R*3*m* with alternating layers of LiO_6_ and NiO_6_ octahedra connected through edge sharing. Reflections were indexed and integrated using the standard *CrysAlisPro* software and refinements were performed using *Jana2006*. Refinements from the superstructure peaks were performed in the standard space group *R*3*m* with varying occupancies of Li and Ni on sites 3*a* and 3*b*. The refinement also confirms that this system exhibits 2 × 2 superstructure with *R*_w_ = 3.54% and unit-cell parameters *a* = 5.8247 (2) Å and *c* = 14.271 (4) Å with a rhombohedral structure. The full crystallographic parameters obtained from refinements are given in Table 1 ▸ and a plot of the unit cell is provided in Fig. 2 ▸. The site occupancies were refined by restricting the total site occupancy and total lithium and nickel content. The site distribution shows a roughly equal composition of Ni ions on both sites, with only small differences between the sites providing the origin of superstructure. The refined thermal displacement parameters indicate that the ions are well localized and show no anomalies.

**Figure 3:**
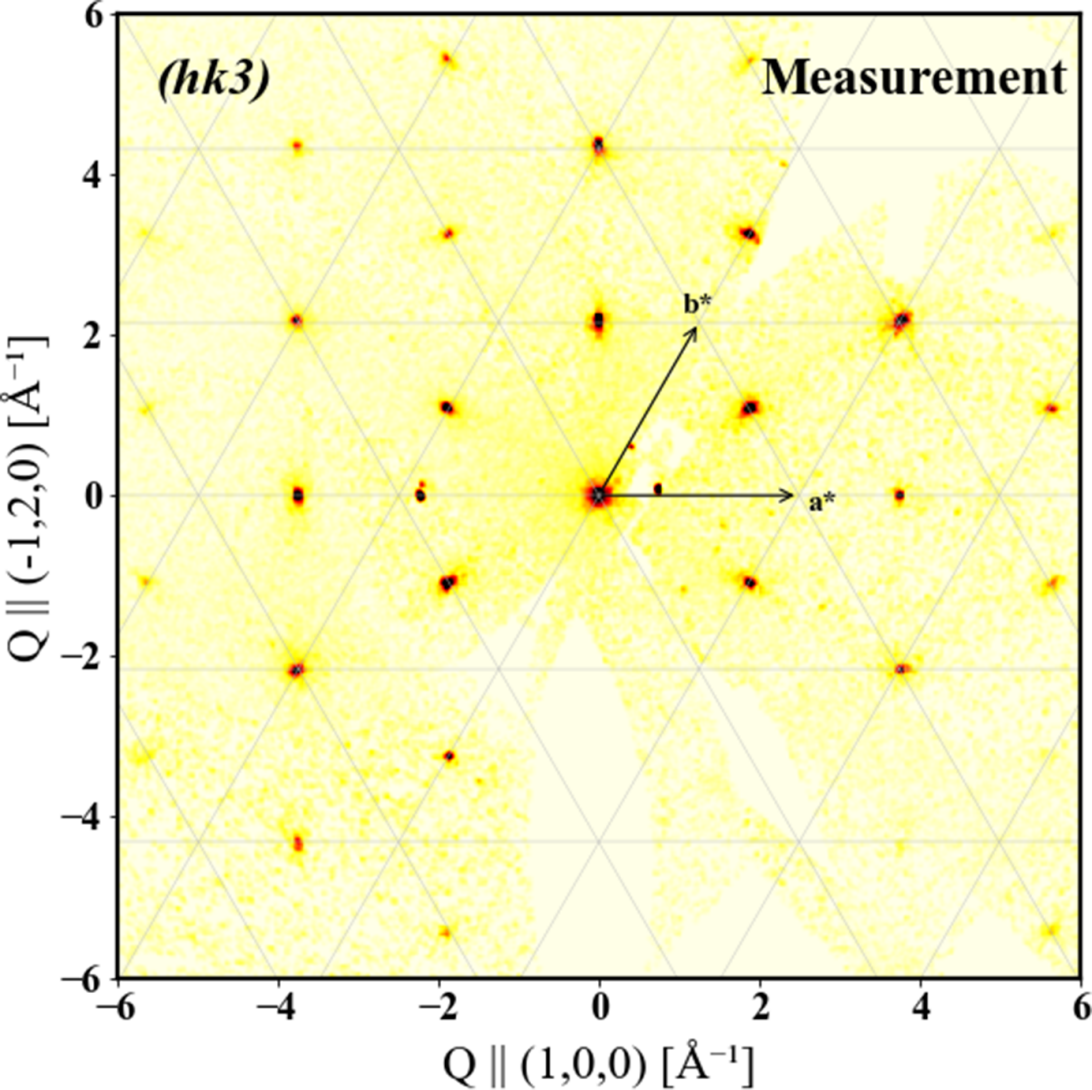
Reciprocal space cuts through the (*hk*3) plane measured by single-crystal XRD.

The diffraction pattern of the (*hk*3) plane is shown in Fig. 3 ▸ with the appearance of clear strong peaks which were identified and indexed according to the unit-cell parameters reported above. The superlattice reflections were detected half way between the principal Bragg reflections, with other small peaks arising from impurity phases.

**Figure 4:**
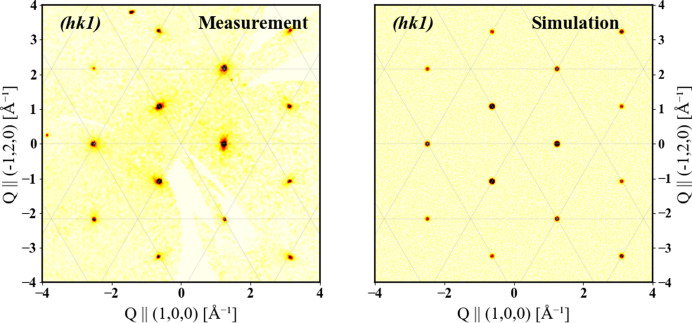
Comparison of calculated and measured reciprocal space maps.

Fig. 4 ▸ shows the comparison of calculated and measured reciprocal space maps at 300 K, which indicates good agreement with the experimental data across all ‘*l’* planes including both the principal and superstructure reflections [shown here (*hk*1)].

**Figure 5:**
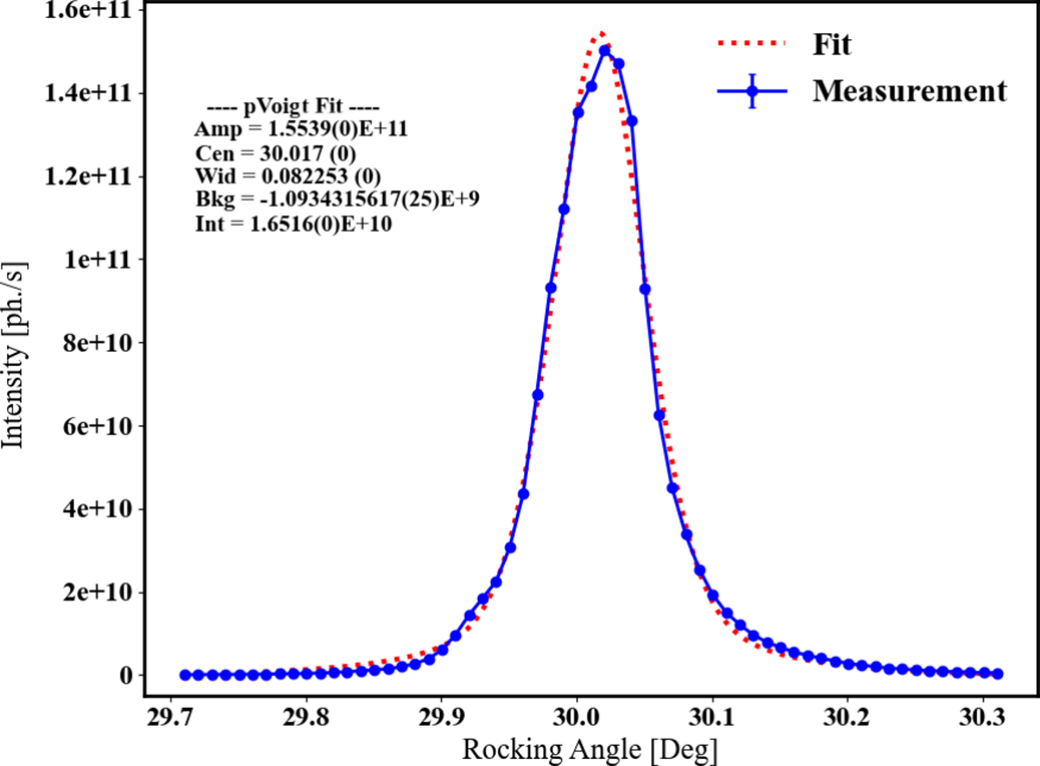
Rocking curve of a principal Bragg reflection (0, 0, 12) with FWHM = 0.08°, indicating the high quality of the crystallite.

A different sample with a flat surface was measured on I16. Fig. 5 ▸ shows the rocking curve of a reflection used for alignment, with peak full width at half-maximum of 0.08° indicating a low mosaic spread. Reciprocal space maps generated by re-mapping detector images into reciprocal space revealed the same superlattice pattern measured with the laboratory diffractometer, with additional reflections appearing halfway between principal reflections in the *L* = odd planes. These results confirmed the propagation vector of τ = (0, ½, 1) observed in the laboratory XRD measurement. Other peaks were also observed in the reciprocal space map but these other peaks do not reappear at symmetric locations. The low intensity of these other reflections suggests that these are from impurity phases. Further analysis of the diffracted intensities should reveal the nature of this superstructure, be it Li or Ni^3+^/^4+^ ordering. Whilst many other small reflections were observed in the reciprocal space maps, these were rejected as superlattice peaks due to either their lack of a resonant signal at the Ni *K* edge, or not being maximized at the sample centre.

Reciprocal space maps at low temperature did not reveal any additional reflections away from the known reflections from long-range magnetic or electronic ordering, however the short duration of the experiment meant that we were not able to use background subtraction methods such as a graphite analyser crystal to look for very weak signals.

**Figure 6:**
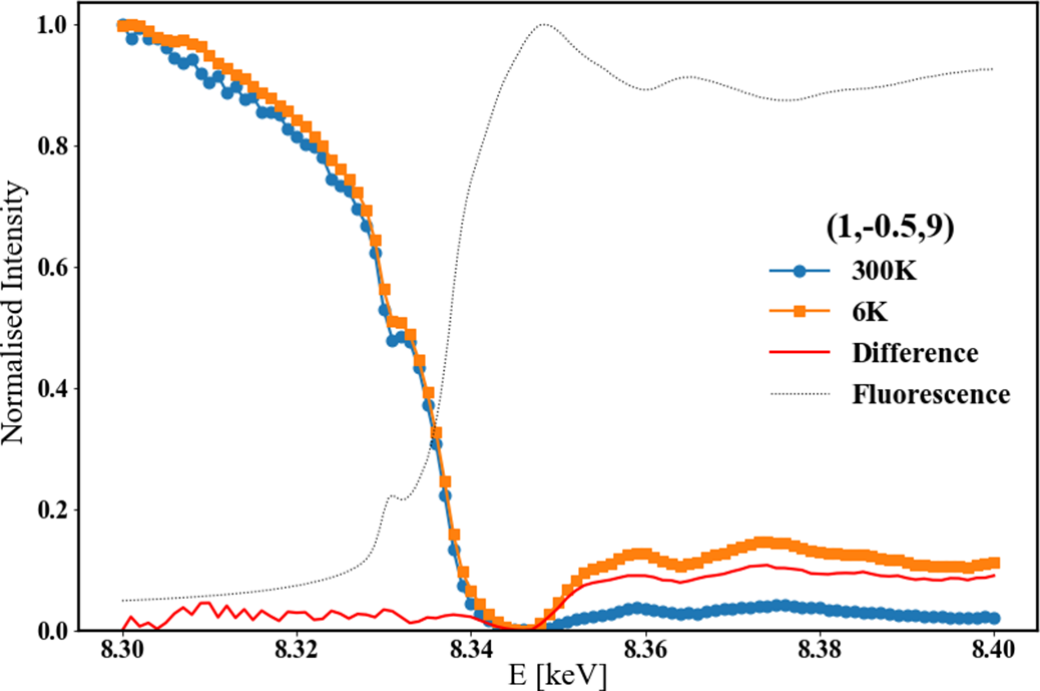
Energy scan of superlattice reflection (1, −½, 9) at 300 K and 6 K, with difference shown in red and background fluorescence shown in black.

Constant-wavevector energy scans were performed on principle reflections and superlattice reflections to look for evidence of resonant components to the scattered intensity of these reflections, which could indicate electronic ordering. An example is show in Fig. 6 ▸. Typically, these energy scans show a reduction in peak intensity through the absorption edge and that was observed in all cases. No resonant behaviour was observed in any reflection, however it is notable that at low temperature, peak intensity above the absorption edge recovers to a higher level than at high temperature, suggesting an increase in short-range interatomic electronic behaviour. The fluorescence measurement, taken at grazing incidence with the detector missing any reflections, shows a Ni *K*-edge line shape that suggests a mixture of nickel oxidation states, however in this experiment it was not possible to accurately determine this mixture.

## 4. Conclusion

In summary, we report a breakthrough in the first single-crystal growth of LiNiO_2_ using the floating zone technique by successfully optimizing the growth conditions. The as-grown single-crystal quality has been thoroughly analysed using laboratory and high-energy X-rays. For the first time we find superlattice reflections with propagation vector (0, ½, 1) which are seen clearly in both the laboratory source and on REXS. The superlattice pattern did not change as a function of temperature which infers a single phase below the ordering temperature. Resonant energy scans at Ni *K* edge reveals no evidence of resonant peaks at principal or superlattice peaks. This implies that LiNiO_2_ system is dominated by structural scattering. As this system is dominated by structural scattering it is believed that there will be some excess Ni^2+^ in the Li^+^ layers. This will significantly alter the magnetic properties and often lead to a spin glass behaviour masking the intrinsic quantum spin liquid behaviour.

## Supplementary Material

Crystal structure: contains datablock(s) global, I. DOI: 10.1107/S2052520626001009/bal5004sup1.cif

Table of superstructure refinement. DOI: 10.1107/S2052520626001009/bal5004sup2.png

Reciprocal space cuts through the (h,k,3) measured by single-crystal XRD. DOI: 10.1107/S2052520626001009/bal5004sup3.png

Comparison of calculated and measured reciprocal space maps. DOI: 10.1107/S2052520626001009/bal5004sup4.png

Structure factors: contains datablock(s) global, I. DOI: 10.1107/S2052520626001009/bal5004Isup5.hkl

CCDC reference: 2527204

## Figures and Tables

**Table 1:** Refinement of LiNiO_2_ at 300 K with 2 × 2 superstructure in space group *R*3*m*

	Site	*x*	*y*	*z*	Occ.	*U*_iso_
Ni2_1	3*a*	0	0	0	0.4364 (7)	0.00606 (8)
Li1_1	3*a*	0	0	0	0.5636 (7)	0.00606 (8)
Ni2_2	9*e*	0.5	0	0	0.4998 (4)	0.00556 (5)
Li1_2	9*e*	0.5	0	0	0.5002 (4)	0.00556 (5)
Ni1_1	3*b*	0	0	0.5	0.5657 (7)	0.00554 (6)
Li2_1	3*b*	0	0	0.5	0.4343 (7)	0.00554 (6)
Ni1_2	9*d*	0.5	0	0.5	0.4995 (3)	0.00556 (5)
Li2_2	9*d*	0.5	0	0.5	0.5005 (3)	0.00556 (5)
O1_1	6*c*	0	0	0.25005 (3)	1	0.0171 (4)
O1_2	18*h*	0.49866 (3)	0.50134 (3)	0.249279 (16)	1	0.0173 (3)
