# Supplementary figures and images for: Unravelling superstructure and electronic ordering in LiNiO2 bulk single crystals grown by optical floating zone technique
Source: Acta Crystallogr B Struct Sci Cryst Eng Mater. 2026 Mar 5;82(Pt 2):154–8. doi: 10.1107/S2052520626001009 (PMC13058902; doi:10.1107/S2052520626001009)

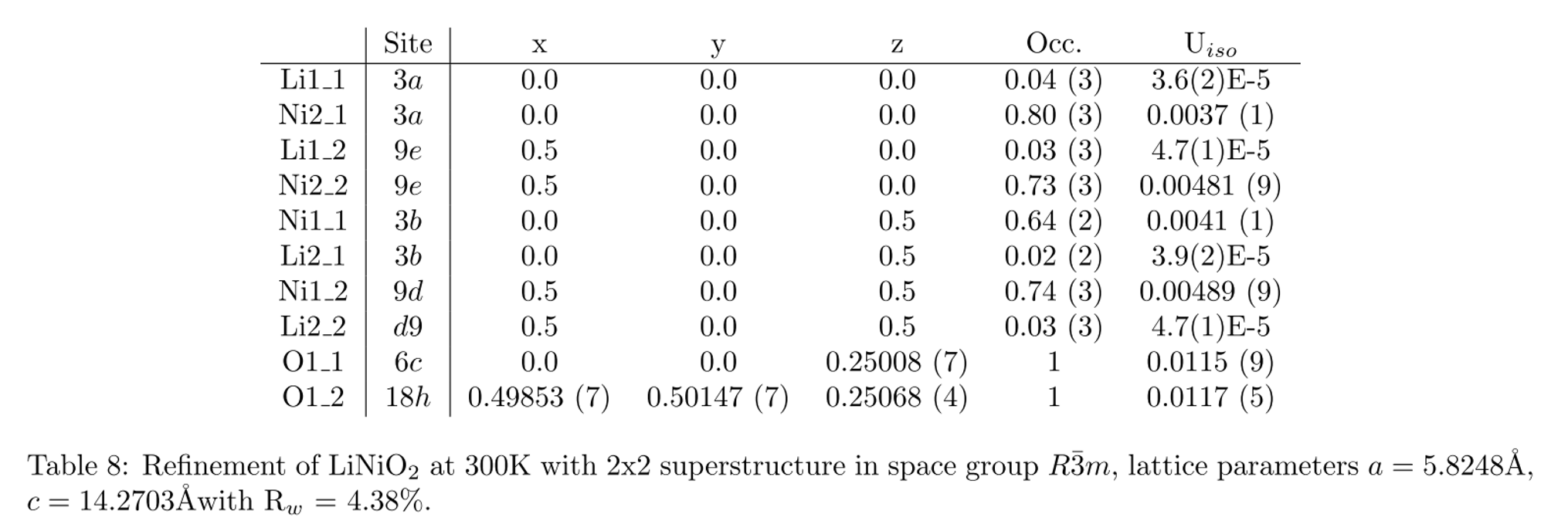

Supplement: Supplementary file 2 [file b-82-00154-sup2.png]

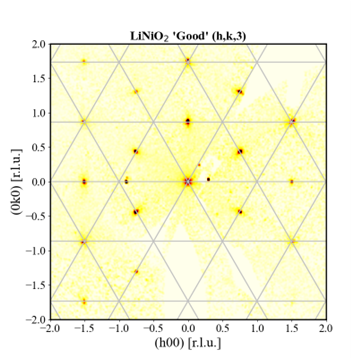

Supplement: Supplementary file 3 [file b-82-00154-sup3.png]

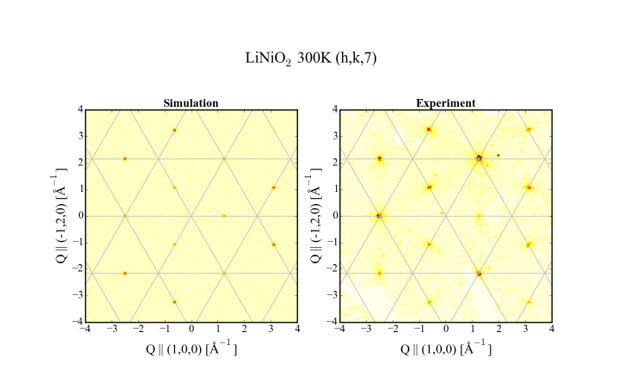

Supplement: Supplementary file 4 [file b-82-00154-sup4.png]
